# Supplementary material for: Nutritional Care of Hospitalized Children in Belgium: A Follow-Up Survey
Source: Nutrients. 2025 Feb 18;17(4):718. doi: 10.3390/nu17040718 (PMC11858114; doi:10.3390/nu17040718)
Supplement: Supplementary file 1 [file nutrients-17-00718-s001.zip › nutrients-3446696-supplementary.pdf]

Accompanying Introduction

First of all, thank you for your interest in completing this survey. Before proceeding to the survey, we are required to ask for your consent to process your data for this study. Furthermore, please note that your participation in this study is entirely voluntary, meaning you can refuse to complete the survey and are free to stop at any time.

We only store personal data (name, hospital email address) necessary to register who has already responded to the survey. Your data will be stored for the duration of the study and the processing of results. If you have any questions or complaints about your privacy, you can contact the Data Protection Officer at gegevensbescherming@uzbrussel.be. Your data will only be used for this research. The study has been approved by the Ethics Committee of UZ Brussel. The committee ensures that the required conditions for your protection and the observance of your rights are met.

- Yes, I agree and wish to participate in the study.
- No, I do not agree and do not wish to participate in the study.

Demographics and Organization

1. What type of hospital are you head of the department in?
    - University
    - Regional
2. How many pediatric beds (excluding PICU/NICU) are there in your department?

    ____

3. What type of medical record system is used in your department?
    - Electronic
    - Paper-based
    - Combination of both
4. Are dietitians employed in/for your department?
    - Yes
    - No
5. How many dietitians, expressed in full-time equivalents (FTE), work in your department? (If the previous answer was "No," please indicate "0.")

    ____

6. Is the dietitian staffing sufficient to implement a timely and high-quality nutrition policy?
    - Yes, almost always
    - Usually
    - Rarely
    - No, (almost) never
7. Do dietitians accompany physicians during patient rounds?
    - Always
    - Often, but not systematically

   - Rarely
   - Never
   - We do not have a dietitian
8. Is there consultation with dietitians?
   - Daily
   - A fixed number of times per week, but not daily
   - Once a week at a set time
   - Ad hoc
   - We do not have a dietitian
9. What are the responsibilities of a dietitian in your department? (multiple answers possible)
   - Organizing and monitoring nutritional screening
   - Conducting nutritional screening
   - Weighing and measuring children
   - Nutritional assessment of malnourished children
   - Monitoring intake of children in the ward
   - Initiating/supervising supplementary feeding
   - Initiating/supervising tube feeding
   - Initiating/supervising TPN
   - Other: ___________
10. Is there a pediatric nutrition team in your hospital?
    - Yes
    - No
11. If yes, who are the members of this team? (multiple answers possible)
    - Dietitian
    - Speech therapist
    - Pediatrician
    - Pharmacist
    - Nurse
    - Psychologist
    - Pedagogue
    - Management/administrative staff
    - Other: ___________
12. Are malnourished children ever admitted to your department?
    - Never
    - <1x/month
    - 1x/month - <1x/week
    - ≥1x/week
13. Is there a protocol for malnutrition in children?
    - Yes
    - No, but under development
    - No
14. What elements are included in the protocol for malnutrition in children? (multiple answers possible)
    - Not applicable (we do not have a protocol)
    - Screening for malnutrition
    - Diagnosis of malnutrition
    - Treatment of malnutrition
    - Follow-up of the malnourished patient
    - Other: ___________

Screening

15. Is malnutrition screened for in your department (in any way)?
    - Yes, systematically
    - Only if there is a clinical suspicion of malnutrition/nutritional problems
    - Sometimes
    - Never
16. How is malnutrition screened for? (multiple answers possible, a question about diagnosis follows later)
    - Based on height/weight
    - Using a screening tool
    - Based on clinical observation
    - Based on blood parameters
    - Based on mid-upper arm circumference
    - Based on skinfold measurements
    - Based on changes in intake
    - Using technical methods such as bioelectrical impedance analysis, DEXA scan, etc.
    - Other: ___________
17. If you use a screening tool, which one is it?
    - STRONGkids
    - STAMP
    - PYMS
    - PNRS
    - PEDISMART
    - A locally developed tool
    - Other: ___________
    - We do not use a screening tool
18. Is the result of the screening (in any form) linked to follow-up?
    - Yes, the patient is referred to a dietitian
    - Yes, the policy is discussed with the attending physician
    - No, there is no standard follow-up linked to the screening
19. Who most frequently signals potential nutritional issues/concerns about nutritional status to the medical team?
    - Parents
    - The child themselves
    - Nurse
    - Dietitian
    - Medical staff themselves
    - Other: ___________

Assessment

20. What are the guidelines for weighing admitted children?

    - Children are weighed daily
    - Children are weighed at set times (as requested by the physician or dietitian)
    - Children are weighed only upon admission and discharge
    - Children are weighed only upon admission and discharge unless they stay for a certain duration

- Children are weighed only upon admission
- Children are weighed only upon admission unless they stay for a certain duration
- We have no guidelines

21. What are the guidelines for measuring the height of admitted children?
    - Children are measured daily
    - Children are measured at set times (as requested by the physician or dietitian)
    - Children are measured only upon admission and discharge
    - Children are measured only upon admission and discharge unless they stay for a certain duration
    - Children are measured only upon admission
    - Children are measured only upon admission unless they stay for a certain duration
    - We have no guidelines
22. Are weight and height systematically plotted on a growth chart/as a z-score?
    - Yes, always
    - Usually, but not systematically (only when a problem is suspected)
    - Rarely
    - Never
23. Who interprets the growth charts?
    - Pediatrician
    - Pediatric resident
    - Dietitian
    - Other: ___________
24. How are children weighed? (multiple answers possible)
    - Age-appropriate scales
    - The weight is provided by the parents
    - Estimated weight based on a formula
    - Estimated weight based on assessment/experience
    - Other: ___________
25. How are children measured? (multiple answers possible)
    - Measuring board for children under 1 meter in height
    - Tape measure placed next to the child
    - The height is provided by the parents
    - Stadiometer
    - Estimated height based on assessment/experience
    - Segmental measurements (e.g., knee height)
    - Other: ___________
26. How is malnutrition diagnosed? (multiple answers possible)
    - Weight/height/BMI percentiles and/or z-scores
    - Subjective Global Nutritional Assessment (SGNA)
    - Blood/serum markers for macronutrients (e.g., albumin, prealbumin)
    - Blood/serum markers for micronutrients (e.g., vitamin and mineral status)
    - Body composition measurements via anthropometry (e.g., mid-upper arm circumference, skinfolds)
    - Visual inspection
    - Measurement of inflammatory parameters
    - Dietary history
    - Medical history

- Strength and energy measurement (e.g., exercise test, handgrip strength)
- Other: ___________

27. Who primarily diagnoses malnutrition?
    - Nurse
    - Physician
    - Dietitian
28. Do you use the flowchart suggested by, for example, the Flemish Association for Pediatrics?
    - Yes (and it works well)
    - Yes, but it does not work optimally
    - No, but we use a comparable flowchart (and it works well)
    - No, but we use a comparable flowchart (but it does not work optimally)
    - No, I see no added value in it
    - No, but I would like to implement it

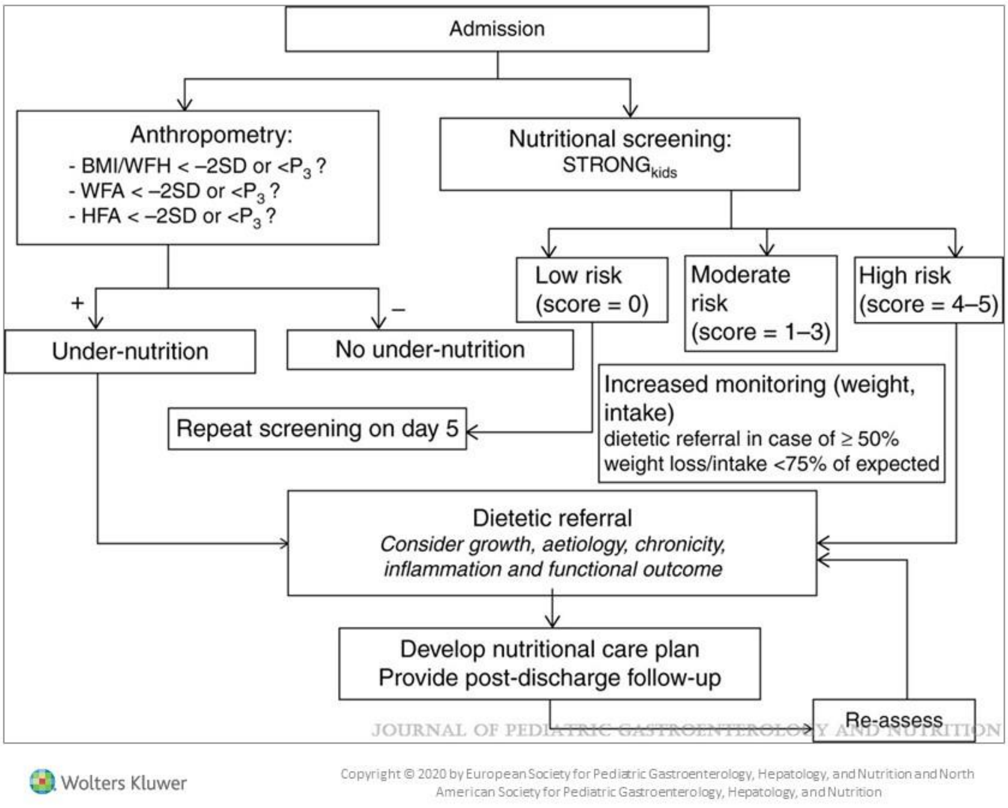

Treatment

29. Is there a protocol for the treatment of malnutrition?
    - Yes
    - No, but under development
    - No
30. What is the most frequent first step in treating malnutrition in your department?
    - Providing additional calories in the diet
    - Starting oral supplementation
    - Starting tube feeding

- Starting TPN
- Other: ___________

31. Is the intake of all children routinely monitored during admission?
    - Yes, by recording/counting calories
    - Yes, by estimating/assessing the percentage of the meal consumed
    - Yes, through meal time audits
    - No, intake is not routinely monitored for all children
32. Is the intake of at-risk/malnourished children routinely monitored during admission?
    - Yes, by recording/counting calories
    - Yes, by estimating/assessing the percentage of the meal consumed
    - Yes, through meal time audits
    - No, intake is not routinely monitored for these children

Follow-up

33. Is information about nutritional status/nutrition policy included in the discharge letter?
    - Yes, always
    - Yes, but only if (temporary) supplementation/TPN was provided
    - Rarely
    - Never
34. If information about nutritional status/nutrition policy is included in the discharge letter, what is mentioned? (multiple answers possible)
    - Weight and height at discharge
    - Which supplementation/tube feeding/TPN was provided
    - The nutritional plan for home
    - Other: ___________
35. If there was a problematic nutritional status or a nutritional intervention during admission, is nutritional follow-up provided?
    - Always, by the physician
    - Always, by the dietitian
    - Always, by both the physician and dietitian
    - Sometimes
    - Never

Barriers/Training

36. What barriers do you experience in practice regarding nutritional screening? (multiple answers possible)
    - Lack of knowledge
    - Lack of time (workload)
    - Lack of staff/resources
    - Lack of motivation among staff (nutrition is not perceived as important)
    - Lack of appropriate equipment
    - There are no significant barriers
    - Other: ___________
37. What barriers do you experience in practice regarding assessing nutritional status? (multiple answers possible)
    - Lack of knowledge
    - Lack of time (workload)
    - Lack of motivation among staff (nutrition is not perceived as important)

- Lack of appropriate equipment
- There are no significant barriers
- Other: ___________

38. What barriers do you experience in practice regarding the treatment of malnutrition? (multiple answers possible)
    - Lack of knowledge
    - Lack of time (workload)
    - Lack of motivation among staff (nutrition is not perceived as important)
    - Lack of appropriate equipment
    - Lack of reimbursement
    - There are no significant barriers
    - Other: ___________
39. Are you interested in a tailored workshop for the multidisciplinary team in your hospital to explore how nutritional care could be optimized?
    - Yes
    - No
40. Would you like support in developing and implementing a protocol in your hospital?
    - Yes
    - No
41. Are you interested in a clinical lecture on nutritional care for the various departments within your hospital?
    - Yes
    - No
42. Do you have any additional comments on the topic of malnutrition/nutritional screening in general that you would like to share because they were not or insufficiently addressed?
    -
